# Self-paced online learning to improve knowledge competencies for hypertension among medical students in Uganda: A pre-post study

**DOI:** 10.1371/journal.pgph.0001609

**Published:** 2023-07-17

**Authors:** Anbrasi Edward, Robert Kalyesubula, George Pariyo, Andrew Peter Kyazze, Xiao Hu, Lawrence J. Appel, Kunihiro Matsushita

**Affiliations:** 1 Department of International Health, Johns Hopkins Bloomberg School of Public Health, Baltimore, Maryland, United States of America; 2 Departments of Medicine and Physiology, Makerere University College of Health Sciences, Kampala, Uganda; 3 Department of Physiology, Makerere University College of Health Sciences, Kampala, Uganda; 4 Department of Epidemiology, Johns Hopkins Bloomberg School of Public Health, Baltimore, Maryland, United States of America; 5 Department of Medicine, Johns Hopkins University School of Medicine, Baltimore, Maryland, United States of America; 6 Department of Epidemiology, Johns Hopkins Bloomberg School of Public Health, and Johns Hopkins School of Nursing, Baltimore, Maryland, United States of America; 7 Department of International Health, Johns Hopkins Bloomberg School of Public Health, and Johns Hopkins School of Nursing, Baltimore, Maryland, United States of America; PLOS: Public Library of Science, UNITED STATES

## Abstract

The growing burden of hypertension (HT) is projected to reach 1.56 billion globally by 2025 and is an increasing public health concern, even for low- and middle-income countries (LMIC) like Uganda, where the prevalence of HT is 31.5%. The objective of this study was to test the effectiveness of a freely available HT online course on knowledge competencies for medical students in Uganda. The online course was developed by a multidisciplinary team at Johns Hopkins University to address HT control in resource-constrained healthcare settings. Students in the 3^rd^, 4^th^, and 5^th^ years of medical school were randomly selected to participate in the online course. Pre and post knowledge tests were administered using an online survey system. Of the 201 invited students, 121 (60.2%) completed the study. Significant improvements in mean knowledge scores were evident following the online course completion for Module 1, Fundamentals of HT (21.9±2.5 to 23.7±2.5, p<0.001), and Module 2, Basics of HT Management (14.9±3.3 to 18.5±4.3, p<0.001). No statistically significant differences were evident by gender or school year. Students who took a shorter duration to complete the course had significantly higher mean score improvement between pre- and post-test (mean score improvement 7.0 if <4 weeks, 3.6 if 4–8 weeks, and 3.7 if >8 weeks, p<0.003). Students recognized information on blood pressure measurement (32.2%) and HT management (22.3%) as the most important concept addressed in the course. A self-paced online course, complementing medical school training, improved knowledge on HT burden and management in Uganda.

## Introduction

The growing burden of hypertension (HT), estimated to reach 1.56 billion globally by 2025, is a major public health concern. In Uganda, national prevalence of HT is 31.5%, which has enormous implications for HT screening and management for a population of 45.7 million [1, 2]. As HT is the most important—and modifiable—risk factor for stroke and heart disease, which are the leading causes of death worldwide, the treatment of HT has substantial benefits. Specifically, lowering systolic blood pressure (SBP) by 10 mm Hg has been estimated to reduce all-cause mortality by 13.3% across all stages of HT regardless of comorbidities [3–6]. Undetected, poorly managed, and uncontrolled HT contributes to the high morbidity and mortality rates of non-communicable disease in Uganda [7]. As the HT burden increases, it is concerning that nine out of ten people (90%) are unaware of their hypertensive condition [7].

### Hypertension control in Uganda

Like most healthcare systems in Sub-Saharan Africa, the Ugandan health system faces multifaceted barriers for the control and management of HT, including chronic health workforce deficits, with the physician density estimated at 0.954 per 10,000 population, based on the 2015 WHO Global Health Observatory data [8]. A cross sectional study in 2 districts showed that 80% of the 126 health facilities were managed by non-medical doctors or non-physician health workers [9]. HT guidelines were available in only 46% of the health facilities, and 58.3% of the health providers reported that they had never seen HT guidelines. Ten percent of the facilities did not have functional blood pressure (BP) measuring devices, and except for 1 facility, none reported calibrating the devices. Stockouts of antihypertensive medicines were reported in 50% of the facilities [9]. Another study of 13 regional referral hospitals, 27 district hospitals, and 13 health centers, showed that there were deficits in essential BP screening equipment and tests for NCD risk factors (>50%), lack of HT clinics (>80%), lack of patient registers (>50%), and lack of HT guidelines (>85%) [10]. A follow up study also illustrated similar deficits for antihypertensive medications and BP screening equipment [11].

### Healthcare provider knowledge on hypertension

Aside from these systemic barriers, poor health provider knowledge, and skill competencies pose additional constraints. An overwhelming proportion of healthcare providers, (98%), reported requiring additional training in HT management [9]. Knowledge gaps on BP thresholds for HT were evident, as 37.9% of diagnosed patients were misclassified as hypertensive at SBP<140mmHg and 14.1% at DBP<90mmHg in a 2015 study. Contrary to the standard protocols for HT management in Uganda, 26% of patients with SBP <140mmHg were prescribed antihypertensives, as were 10.9% for patients with DBP <90mmHg. Inadequacies in knowledge competencies were also reported in another study where 75% of the clinical officers and 31% of medical doctors felt their clinical training did not prepare them adequately to manage HT in patients [10].

Equipping future physicians with optimal knowledge and skill competencies is of paramount importance for effective HT control. The Government of Uganda in partnership with a Non-Governmental Organization (NGO) adopted the World Health Organization Package of Essential Non Communicable Interventions and HEARTS training program [12, 13]. Government or NGO funded seminars offer refresher courses following training on non-communicable disease management, through continuous medical education. The HT guidelines developed by WHO for low and middle income countries integrate risk assessment, patient education on diet, weight gain, and other lifestyle changes [12]. A pilot study using a multimodal HT educational program integrating the WHO-International Society for Hypertension guidelines for nurses in a Ugandan outpatient clinic showed significant improvements in the outcome measures of knowledge, skills, and attitudes [12, 14].

The medical and nursing school curriculum in Uganda provides basic knowledge on HT and clinical management. However, this is often limited to a single lecture and bed side teaching during rotations in internal medicine and pediatrics, with minimal evaluation mechanisms for determining physician knowledge and skill competencies on HT. Pre-service medical education is often constrained by many barriers including limited time devoted to HT, limited space for outpatient learning, lack of availability of experts, and the substantial burden of co-morbidities [15–18]. The recent Covid-19 pandemic has also exacerbated the challenges due to additional restrictions to onsite learning with frequent university closures, necessitating alternative online options for training [19, 20].

The increasing trend for medical schools to compliment classroom learning with online courses holds great promise but also raises key questions related to knowledge enhancement and perceived value of the course. Online learning platforms have a broader reach for health personnel than traditional training paradigms, where face-to-face encounters are limited by time, space, and cost constraints. This is especially true for resource-constrained settings, where chronic workforce deficits prevail. Freely accessible mobile training innovations for self-paced learning to communicate evidence-based HT clinical standards have shown to improve knowledge competencies of healthcare providers [21].

In recognition of the value of online learning, the Makerere University College of Health Sciences in Uganda integrates a problem-based learning curriculum for self-directed learning with online resources as a source of training [22–25]. One cross sectional study of 180 medical students in Uganda showed that HT is a concern, even amongst medical students, as 14% had elevated SBP [26].

The advancement of training technologies offers unique opportunities for online learning to enhance clinician skills and contribute to reducing diagnostic errors and improving HT management. With the support from the Resolve to Save Lives Initiative, a global initiative aiming to reduce 100 million deaths due to cardiovascular disease, a team at Johns Hopkins University developed a free online HT course on fundamentals of implementing HT programs in resource-constrained settings. The course was primarily designed for program managers and implementers who have oversight of HT control programs but has been regarded as suitable for frontline healthcare providers. Recognizing the evidence of gaps in medical school curricula and poor HT knowledge competencies of healthcare providers, this study was designed to evaluate the effectiveness and perceived value of the online HT course in improving the knowledge competency of medical students in Uganda.

## Methods

### Ethics statement

Ethical clearance was obtained from both Makerere University (IRB # SBS-800) and the Johns Hopkins Bloomberg School of Public Health (IRB # 13116). A formal online consent form approved by the Johns Hopkins and Makerere University IRB was used to obtain informed consent from all study participants. The consent form included information on the voluntary nature of the study, the option to drop out of the study at any time, and to refrain from answering any question if they felt a level of discomfort.

Standard procedures were followed to ensure participant confidentiality. The first email invitation to join the study was sent by the Johns Hopkins Principal Investigators using a standard script describing the study purpose, and online consent was obtained from the medical students through the REDCap software. This was followed by another email invitation by a Makerere University research assistant who was a recent graduate of the medical school. If the selected participant failed to respond to both invitations, the Makerere university research assistant followed up with a phone call, WhatsApp message, or Short Messaging Service (SMS). The research assistant’s role was to determine the reason for non-response and clarify any participant concerns. Neither the research assistant, the Makerere principal investigator, nor the academic registrar had access to the test scores, to ensure privacy of the participants. The online pre-post test scores for the HT course were not intended to be factored into the student’s academic assessments. Additional precautions were taken to ensure participant privacy.

### Study design and sampling

The study was conducted jointly by the Johns Hopkins Resolve to Save Lives team and the Makerere University College of Health Sciences in Uganda. The HT online course was designed by a multidisciplinary team at Johns Hopkins and is available at https://globalhypertensionathopkins.org/courses/hypertension. The course is comprised of six modules: 1. fundamentals of HT disease, 2. basics of HT diagnosis and treatment, 3. clinic-based management, 4. community-based management, 5. medication supply chain systems, and 6. improving operational effectiveness of HT programs. The first two modules were selected for the study, as they address the basic epidemiology and fundamentals of HT diagnosis and management. Module 1 is comprised of the basic concept of HT, global burden of HT, treatment of HT, reduction of HT-related complications, major challenges of HT management, and HT control programs. Module 2 includes sections on measuring BP, devices to measure BP, HT diagnosis, and treatment options for HT. The course has not been evaluated among healthcare personnel or medical students in a low- and middle-income countries (LMIC) setting. Therefore, we designed the study to evaluate the effectiveness of this self-paced online course, which compliments the classroom teaching provided to medical students.

A pre-post study design was used to measure differences in knowledge scores on HT among medical students. The Makerere Medical School offers a 5-year program with an internship, and students were selected from the third, fourth, and fifth years of medical school, as years 1–2 are focused on pre-clinical training with basic sciences and years 3–5 are focused on clinical instruction for medical schools in Uganda. An initial sample size of 93 students, 31 in each year of medical school, was estimated to detect differences in knowledge scores, for a two-tailed test (at α = 0.05 and 80% power), assuming an effect size of 0.50. Factoring for the design effect of 1.5 and a 30% non-response rate, the final sample size was estimated at 201 students. Employing stratified random sampling, 67 medical students were selected from the third, fourth, and fifth years of medical school from the University register. All selected participants were above 18 years of age.

The invitation to all 201 medical student participants was sent in December 2020. Participants who consented were administered an online 45-minute, knowledge pre-test comprised of multiple choice and true/false questions, using the REDCap platform (https://redcap.jhu.edu/). The 45-minute pre-test was the estimated average time it would take for the participant to complete the test. The test was not programmed to time out after 45 minutes.

If participants did not respond, a follow up reminder was generated automatically through the REDCap system and therefore recruitment occurred over a period of 6 months. Pre-tests were administered right after the participants consented, but participants had to click on the link to take the pre-test. If they consented and did not take the pre-test, the REDCap system generated additional reminders to complete the pre-test. Following completion of the pre-test, participants received a link to view the first two modules of the online HT course. Though each module was paced for approximately 1-2h, the participants had the option of self-pacing and could view the modules multiple times. Two weeks after the completion of the pre-tests, participants received a follow-up invitation with a link to complete the 45-minute post-test. We selected 2 weeks between pre and post-test to ensure retention and eliminate recall issues. It was not based on cited evidence.

As in pre-test, reminders were automatically generated if they failed to take the post tests. The question bank for both the pre- and post-tests were identical, except that the post version included additional questions related to the students’ perception of the online course.

The pre and post tests were conducted remotely through a web-enabled smartphone, PC, or tablet using the REDCap platform. There was no camera enabled monitoring to determine the identity of the participant who took the test, as the test was taken either on a smart phone or a public internet service center or at the university, since it was during the COVID-19 pandemic. However, we took the necessary precautions to generate a unique ID and hyperlink that was only available to the participants to access the pre and post-tests.

The tests were programmed for one sitting. However, due to unreliable internet access, participants who were unable to complete the test were identified by the system administrator. A random computer-generated access code was emailed to the participant to resume the test. To mitigate the risk of gaming, the participant could only resume the test for unanswered questions and did not have the option of revising previously answered questions. Participants who reported being unwell were either excused from the study or given an option to take the post-test within 2 weeks. Due to frequent university closures related to COVID19 and poor internet access, participants were offered a one-time $15 internet access incentive upon study completion.

### Data analysis

Descriptive analysis was performed with de-identified data to assess differences between medical student cohorts. Statistical significance of differences in pre-post knowledge scores was tested using paired 2 tailed t-tests. The comparison of mean pre-post difference between male and female was tested using two-sample t-test. The comparison of pre-post difference between medical students of different year was tested using ANOVA. We also used a linear regression to explore the predictors of knowledge score improvement. We adjusted for sex, year at medical school, the time length between finishing pre-test and finishing post-test, and pre-test score. Due to the limitations of IRB to use personal identifiers, age of the participant was not included in the deidentified data used for data analysis. However, the average age and range was reported for the descriptive information of the participants included in the study.

### Inclusivity in global research

Additional information regarding the ethical, cultural, and scientific considerations specific to inclusivity in global research is included in the S1 Checklist.

## Results

**Fig 1:**
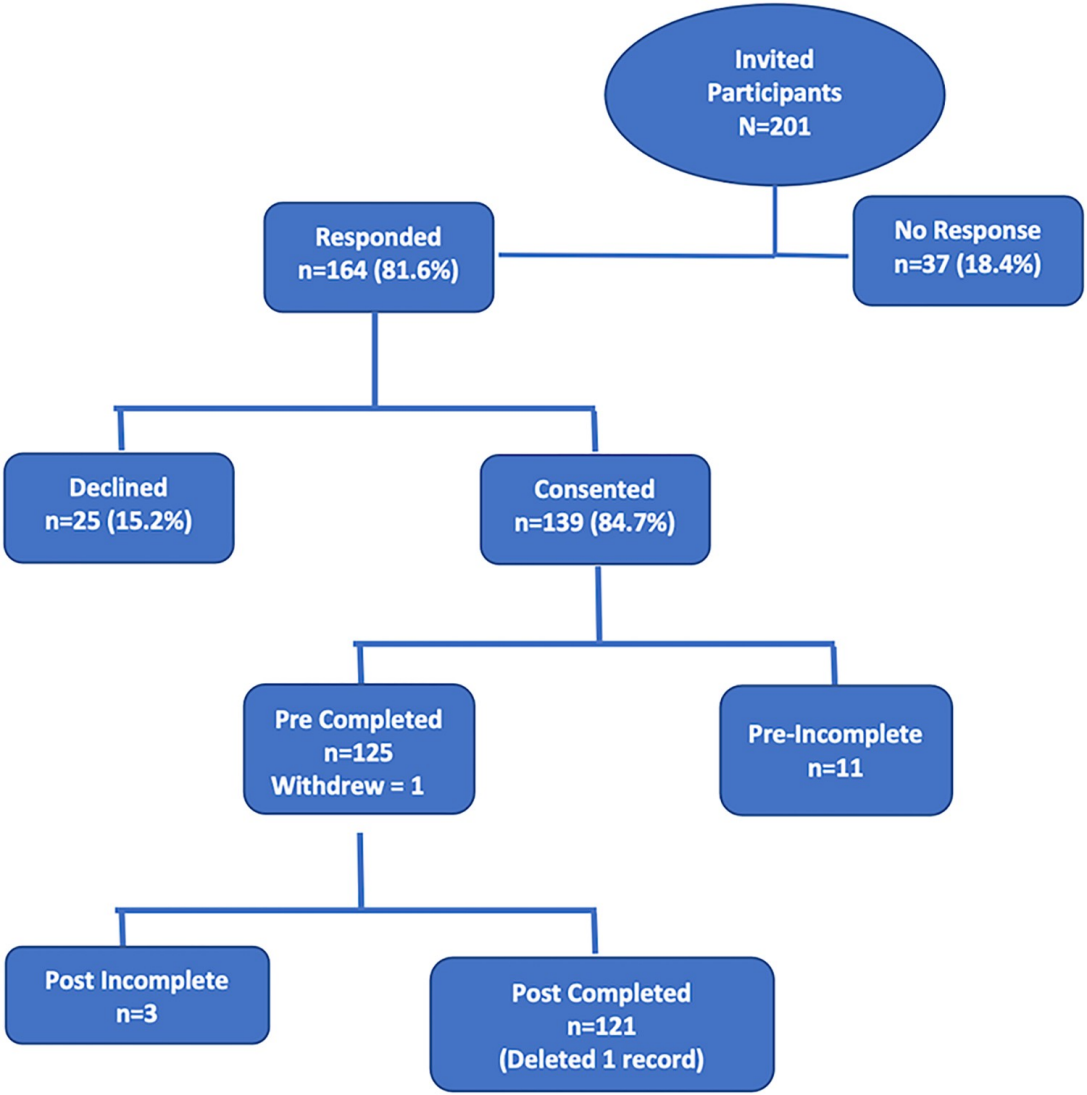
Participant study acceptance and test completion status.

Of the 201 medical students who were invited to participate in the online training, 164 (81.6%) responded, and 139 (84.7%) consented to the study ““Fig 1, Participant Study Acceptance and Test Completion Status””. Most common reasons for declining to participate were lack of time due to competing medical school demands, lack of interest in an online course, and limited or no internet access (15.2%). Of the 139 who consented, 126 students completed the pre-tests and participated in the online training, and 122 (96.8% of 126) completed the post-tests; one record was eliminated as the profile was incomplete.

The final sample included in the analysis was 121 students, 64.5% male and 35.5% female (Table 1). Mean (range) age of the students was 25.0 (20 to 41) years. Twenty-four percent of the participants were in the third year of medical school, 29.8% in the fourth year, 38.8% in the fifth year, and 7.4% had completed the course requirements.

**Table 1:** Characteristics of study participants (N = 121).

Characteristics	N(%)
Sex	
* Male*	78 (64.5%)
* Female*	43 (35.5%)
Age (Data available for 113 participants)	
* Range*	20-41y
* Mean(*±SD)	25.0±3.5y
Year at Medical School	
* Year 3*	29 (24.0%)
* Year 4*	36 (29.8%)
* Year 5*	47 (38.8%)
* Recently Completed*	9 (7.4%)

The study was initially planned to be completed within 4 weeks. However, due to multiple disruptions resulting from COVID-19, the duration between finishing pre-test and finishing post-tests ranged from 2 to 27 weeks ““S1 Fig, Length of Time between Pre and Post Test””.

Gender and year at medical school differences were evident in length of time spent in completing the course and post tests. Male students spent a significantly shorter duration (6.5 weeks vs 9.7 weeks, p = 0.03) to complete the online course than female students ‘“S2 Fig, Length of Time for Study By Gender,””.

Mean duration to complete the course was 6.3 weeks for 3^rd^ year medical students, 8.0 weeks for 4^th^ year students, 7.4 weeks for 5^th^ year students, and 11.7 weeks for those who had completed medical school; differences among these groups were not statistically significant ““S3 Fig, Length of Time for Study By School Year””.

**Fig 2:**
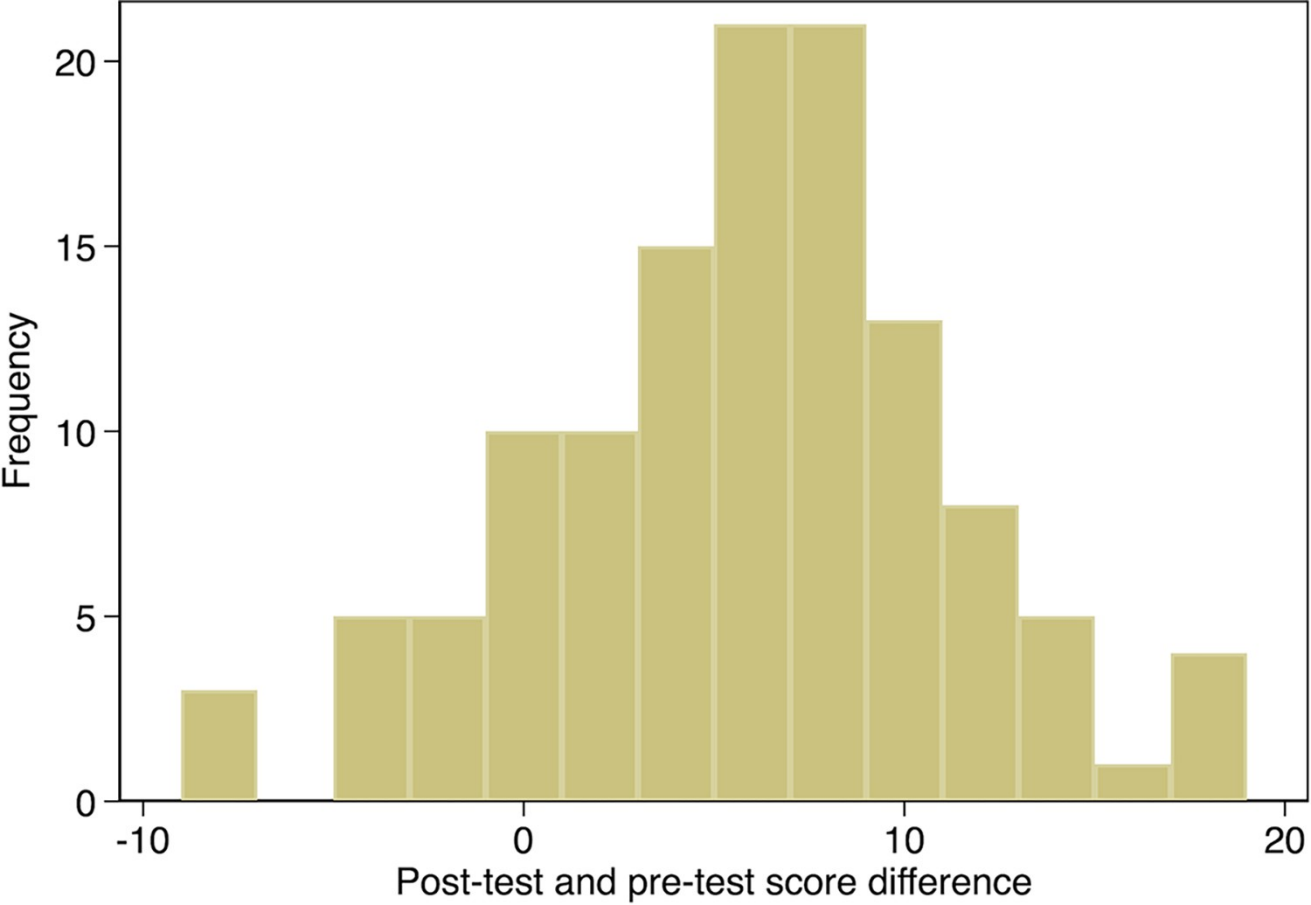
Distribution of pre-post score differences.

**Fig 3:**
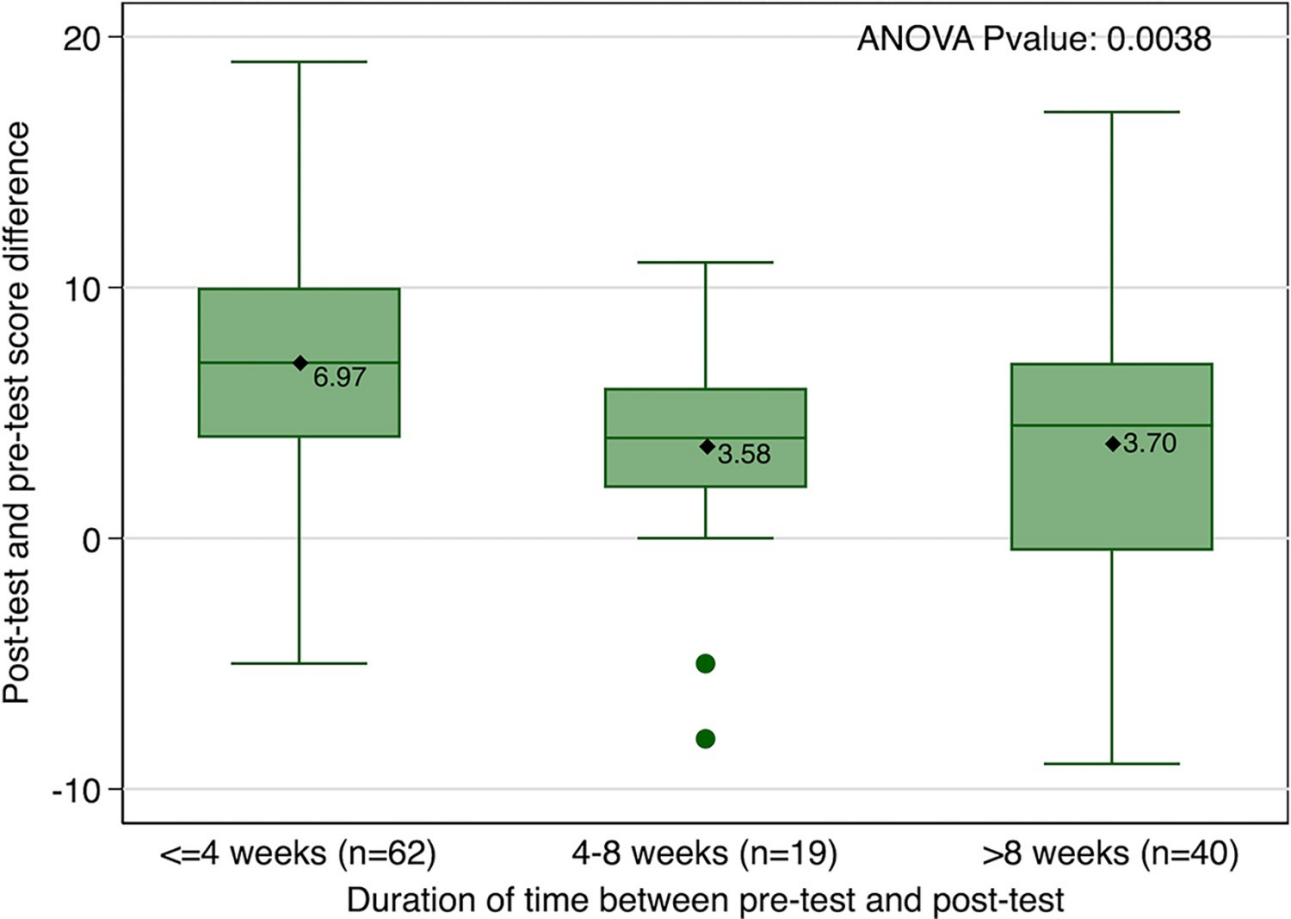
Duration of time pre to post test.

Pre-post knowledge scores for both modules and for the 9 topics covered in the two modules indicated a significant improvement except for HT diagnosis (Table 2). The total knowledge score for both modules 1 and 2 increased by 5.4 points, from 36.8 to 42.2 (p<0.001) following the online course training. However, 19 (15.7%) of the students scored lower in the post-test than the pre-test “”Fig 2, Distribution of Pre-post Score Differences””. Pre-post difference was higher for Module 2 (3.61) than Module 1 (1.74) ““Fig 3, Duration of time Pre to Post Test””.

**Table 2:** Hypertension knowledge scores by Module.

Modules	Maximum Score	Pre-test score (mean ± SD)	Post-test score (mean ± SD)	Post-pre difference	p-value^a^
***Module 1 Fundamentals of HT***					
Concepts of HT	6	4.37±0.80	4.83±0.89	0.45	<0.001
Treatment of HT	5	3.91±1.02	4.49±0.63	0.58	<0.001
Reduction of HT-related complications	4	2.78±0.86	3.17±0.87	0.39	<0.001
Challenges in HT management	4	3.69±0.62	3.83±0.51	0.14	0.006
HT control programs	8	7.17±0.95	7.35±0.93	0.18	0.044
Total score Module 1	27	21.91±2.52	23.65±2.51	1.74	<0.001
***Module 2 Basics of HT Management***					
Measuring blood pressure	9	5.21±1.35	6.21±1.50	0.99	<0.001
Devices to measure blood pressure	2	0.83±0.57	1.29±0.72	0.46	<0.001
HT diagnosis	2	1.36±0.66	1.39±0.64	0.02	0.711
Treatment options for HT	15	7.14±2.07	8.96±2.28	1.82	<0.001
Total score Module 2	28	14.90±3.30	18.51±4.25	3.61	<0.001
***Total score Module 1 and 2***	55	36.81±4.75	42.17±6.11	5.36	<0.001

^a^ Paired t tests

**Fig 4:**
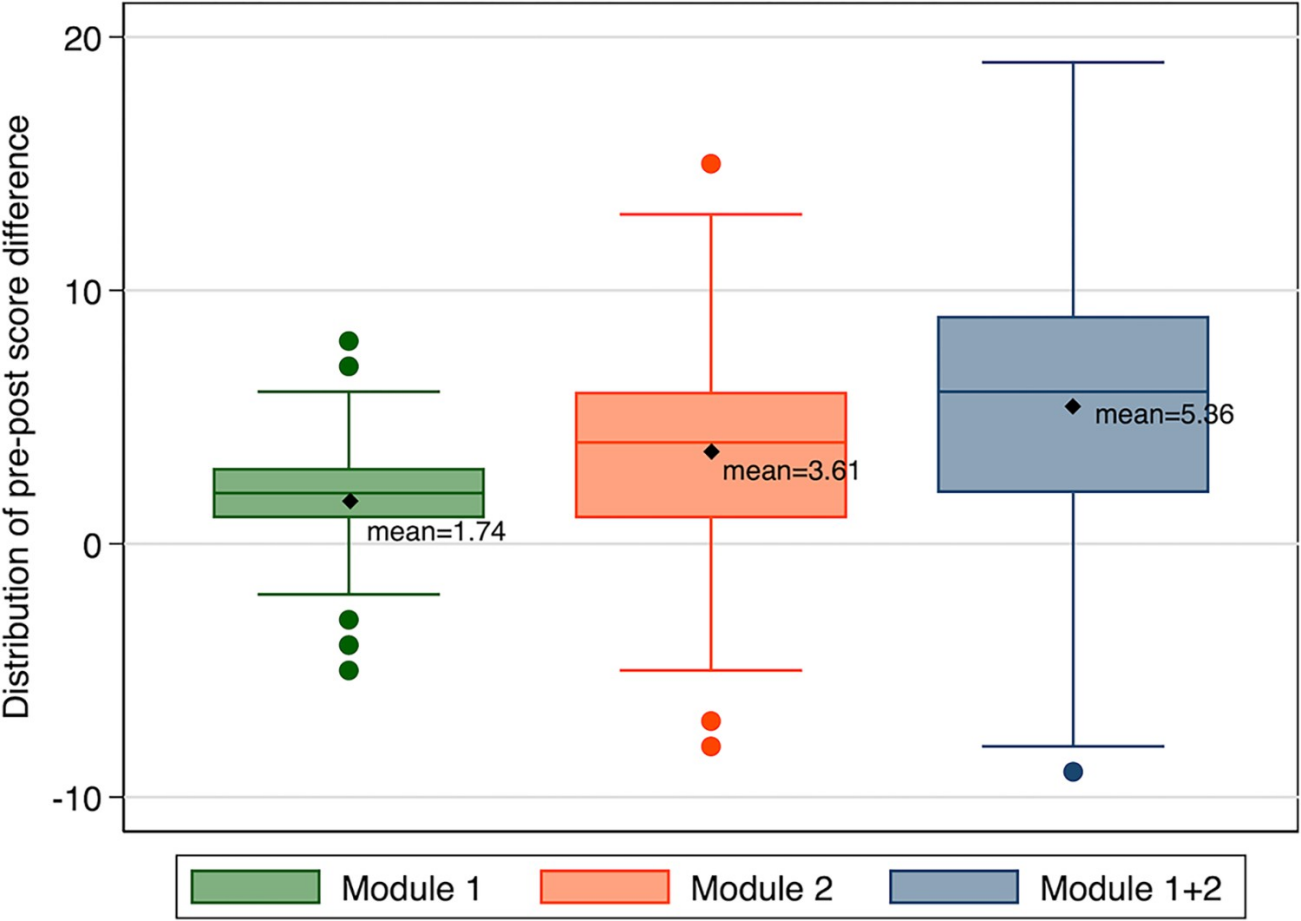
Distribution of pre-post test difference by Module.

There was a significant association (p = 0.004) between the length of time to complete the online course and mean post test scores “‘Fig 4, Distribution of Pre-post Test Difference by Module’”, such that medical students who took the shortest time (<4 weeks) to complete the course had the highest mean improvement in mean test scores (6.97 points).

Significant improvements on specific knowledge components were evident for HT definition, symptoms of HT, and global burden of HT, for the basic concepts of HT (S1 Table). Likewise, knowledge scores for HT treatment showed significant improvements following the online training (treatment with medication, type of antihypertensive medications, and patient education on antihypertensive medications). Knowledge on HT complications also showed improvements for risks of uncontrolled HT, and overall risk for high SBP and/or DBP. Knowledge of HT control programs showed significant improvements for patient counseling by community health workers (CHW) to support patient medication adherence and HT definition for populations at risk. Decline in scores was evident for some concepts, though only the outcome of effective HT control programs reached statistical significance.

Knowledge scores for concepts in Module 2 showed similar differences (S1 Table). Significant improvements were evident for measuring BP, patient preparation standards prior to BP measurement, patient texting messages on phones causing errors in BP measurement, standards for multiple BP measurements, and precautions to be considered prior to BP measurement. Likewise, advantages of automated BP devices, along with terms used for automated and aneroid devices, showed significant improvement in knowledge scores following the training. On the other hand, knowledge of the protocol for a return visit for a patient with elevated BP declined significantly. Treatment options for HT showed significant improvements for several concepts following the online training. These included antihypertensives that did not require laboratory monitoring; standard protocol for determining the type of BP medication; protocol for all types of first line BP medication; first line treatment options; BP lowering medication options; safety, and risk of side effects of BP medicines; treatment options in settings with no laboratory facilities; and reason for automated device recommendation for low resource settings.

With regard to gender differences, the mean increase in the knowledge score was 5.5 for male students and 5.1 for female students (not significantly different) (Table 3). For medical students enrolled in the third year, improvements in knowledge scores were 7.0; 5.3 in 4^th^ year students; 4.6 in 5^th^ year students; and 4.2 in recent medical school graduates; however, improvements were only significant for knowledge on reducing HT related complications (p<0.03).

**Table 3:** Pre-post total knowledge scores by gender and year at medical school.

Participant Gender and Year at Medical School	Pre-test score (mean ± SD)	Post-test score (mean ± SD)	Post-pre difference	p-value
Gender				
*Male*	36.99±4.01	42.47±5.60	5.49	0.73^a^
*Female*	36.49±5.90	41.60±6.97	5.12	
Year at Medical School				
* Year 3*	37.48±3.24	44.48±4.72	7.00	0.28^b^
* Year 4*	36.78±4.15	42.08±5.88	5.31	
* Year 5*	36.74±6.05	41.34±6.68	4.60	
* *Recently completed	35.11±3.30	39.33±6.44	4.22	

^a^ two sample t tests

^b^ ANOVA

Shorter duration of time between the pre- and post-test was significantly associated with increased knowledge scores. Medical students who completed the post-test within 4 weeks had a significantly higher mean score (6.97) than those who took more than 8 weeks to complete the test (3.7) ‘“Fig 3, Duration of time Pre to Post Test’”. Pre-post difference was higher for Module 2 (3.61) than Module 1 (1.74) ‘“Fig 4, Distribution of Pre-post Test Difference by Module’”.

The linear regression results showed that pre-test score was a significant predictor of improved knowledge scores (Table 4).

**Table 4:** Multivariable analysis of factors associated with knowledge scores.

outcome: pre-post score difference*	Coefficients	95% CI		P value
Sex (Ref: female)				
* *Male	-0.34	-2.32	1.64	0.732
Medical status (Ref: Year 3)				
* *Medical student—Year 4	-1.73	-4.27	0.81	0.181
* *Medical student—Year 5	-2.56	-4.95	-0.17	0.036
* *Recently completed medical school	-2.84	-6.78	1.11	0.157
Pre-post duration (unit: week)	-0.21	-0.34	-0.07	0.003
Pre-test score	-0.45	-0.65	-0.25	<0.001
Intercept	25.34	17.17	33.51	<0.001

*Post minus pre test score.

Evaluations of the value of the online course indicated that 99% of the participants felt the course was good or excellent, and there was universal agreement that the learning objectives were achieved (Table 5). Information on BP measurement (32.2%) and HT management (22.3%) were considered the most important elements in the modules. Frequently mentioned recommendations for enhancing the course included additional information on medications, side effects, administration of medicines, doses, and customized treatment plans for patients. Other recommendations included integration of more clinical case vignettes, including more images and videos, information on HT management, reducing the number of quiz questions, using different test questions for pre and post tests and shortening the duration of the course modules.

**Table 5:** Medical student perspectives on online hypertension course.

	N = 121
Overall perspective of training modules	n(%)
* Excellent*	79 (65.3%)
* Good*	41 (33.9%)
* Fair*	1 (0.8%)
Perspective on quiz questions	
* Excellent*	53 (43.8%)
* Good*	61 (50.4%)
* Fair*	7 (5.8%)
* Poor*	-
Perspective on achieving course learning objectives*	
* Strongly agree*	62 (51.2%)
* Agree*	58 (47.9%)
* *Specific area of satisfaction/interest	
* Blood Pressure Measurement*	39 (32.2%)
* HT treatment*	8 (6.6%)
* HT burden*	8 (6.6%)
* HT management*	27 (22.3%)
* HT control programs*	2 (1.7%)
Recommendations for improving HT course	
* Provide more information on medication (side effect*, *administration*, *types and combinations*, *dose*, *and customized treatment regimen for specific patients)*	30 (24.8%)
* Include more clinical cases*	3 (2.5%)
* Include more knowledge on HT management*	6 (5%)
* Quiz questions were too long*	4 (3.3%)
* Too many quiz questions*	6 (5%)
* Use different questions for pre and post test*	2 (1.7%)
* *Shorten Modules	4 (3.3%)
* *Include more video or images	5 (4.1%)
* *Insert page number for the tests	1 (0.8%)

*n = 120 (I record missing)

## Discussion

In this study that enrolled medical students and recent graduates from a medical school in Uganda in an online course on HT competencies, there was overall improvement on knowledge scores following the online training, with variations in knowledge score improvements for elaspsed time between pre and post tests and year at medical school. The findings provide some evidence of the potential of online education even in resource-constrained settings. It is unlikley that the medical school training during the pre-post period influenced the knowledge scores, as prior review of the medical curriculum indicated very minimal information on HT, except for diagnosis and treatment. The students receive one scheduled medical lecture on HT in the third year and it is not comprehensive.

The importance of these findings is underscored by the enormous challenge in Uganda of addressing HT control, as low levels of screening, treatment, and control pose a major public health problem. However, further investigation could delve into variations of learning needs by year of medical school, as well as performance of students in different years of medical school, neither of which was specifically addressed in this research.

Gender differences in the online course completion also highlight the need to determine appropriate gender-sensitive training modalities to ensure equity in learning. We speculate that the engagement of female students in domestic responsibilities during the lockdown might be a factor for poor scores.

Baseline HT knowledge assessment of the medical students enrolled in our research study indicated low scores for basic concepts of HT, its treatment and treatment options, reducing HT related complications, and measuring BP. These findings underscore the need for complimentary learning options to ensure optimal knowledge competencies for HT before the students’ screen and manage patients in the healthcare systems. In an earlier study of healthcare providers, 98% requested additional training in HT management, and significant knowledge gaps were evident with regard to HT thresholds and prescribing antihypertensives: 76.3% agreed that most patients with HT are asymptomatic, 12.2% neither agreed nor disagreed, and 11.5% disagreed [9]. Only 18.1% of the healthcare providers reported receiving additional training for HT management in the two years preceding the study. Although the participants had high levels of confidence regarding correct procedures for BP measurement, they had low confidence levels with regard to counseling patients about diet and lifestyle; prescribing antihypertensives for patients diagnosed with HT; calculating BMI; and managing HT for patients with stroke. Another follow up study showed similar gaps: 75% of the clinical officers and 31% of the medical officers (M.D. with 1y of post-graduate generalist training) felt their clinical training did not prepare them adequately to manage HT in patients [10].

Aside from the Uganda Clinical Guidelines, there are no standard training and educational resources for HT management for physicians at the hospital and health facility level [27]. The Uganda Guidelines provide information on HT measurement, diagnosis, classification, risk factors, symptoms, compliance, investigations, management, addressing HT emergencies, advice on lifestyle modification (diet, cessation of alcohol drinking and tobacco smoking, and weight reduction) for mild HT in the first three months or administration of a thiazide diuretic if not controlled, and treatment for moderate or severe HT. The guideline also provides additional recommendations on management of patients with HT complications such as stroke, heart failure, kidney disease, post-myocardial infarction, coronary artery disease and diabetes. However, the guidelines do not specifically address standards for patient preparation prior to BP measurement, correct step by step procedures for measuring BP by manual or automated devices. Low or no-cost certification training programs for resource-constrained environments, such as the one used in this study, have been recommended to achieve optimal performance for healthcare providers for clinical management of HT [28].

The challenges reported by medical students such as unreliable internet connection would equally affect in-service personnel and program managers. Health planners contemplating widescale adoption of online learning for healthcare providers will need to work out strategies through which connection charges are eliminated or otherwise mitigated. The challenge of lack of time may be less significant for in-service health personnel undertaking the online course as part of a required continuous professional development program as they presumably will be able to allocate time. Despite the lack of reliable internet availability, the access to computers and smart technology by medical personnel is increasing, presenting opportunities for information and knowledge acquisition. Contextually designed e-learning platforms offer affordable opportunities to medical professionals for optimizing self-paced learning in contexts where prospects for continued medical education are limited.

The online course was designed for program managers in low resource settings. However, the study on the effectiveness of the course in improving knowledge among medical students provides some evidence of a wider applicability of the online course in resource-constrained settings. The effectiveness of the online course in improving knowledge on HT among medical students offers promise for improving HT knowledge competencies for healthcare providers. This will need to be evaluated further by conducting studies targeting in-service personnel. The health professionals’ accreditation authorities require annual evidence of continuous professional development courses. In the Ugandan context where in-person training often involves travel away from the point of service, an online course, available to healthcare providers with internet access, helps to bridge the gap in knowledge at a potentially lower cost. The importance of such a program for in-service health workers is growing given the rising awareness of the burden of HT and other non-communicable diseases in Uganda and other low resource settings. The associated costs for establishing these online resource capabilities for healthcare providers and program managers warrants further investigation.

Over 95% of the medical students who participated in the study found the course very useful, providing evidence in support of online learning. The slow uptake and lack of participation from some of the students is typical of the behavior change in the learning curve that will need to be addressed before online learning and self-paced studying becomes a norm. The study was conducted at the beginning of the pandemic, during a time with many uncertainties. Applying Kirkpatrick’s evaluation model for measuring reactions, learning behavior, and results, the online course can be appropriately evaluated to determine the effectiveness of knowledge and skills, building the potential to improve HT control programs in LMIC settings that have limited training resource opportunities [29].

As a consequence of chronic physician workforce deficits, nurses and midwives play major roles in patient care, including screening, making vital observations, and instructing patients [30]. Moreover, the educational role of nurses has been expanded and now provides a major contribution to patients’ improved lifestyle behaviors, physical activity, weight management, stress relief, alcohol intake, medication adherence, and self-efficacy (30). The online course may be a vital learning tool to enhance the competencies of nurses, NCD coordinators, and even community health workers who are also at the forefront of screening and follow-up of patients.

Online learning offers feasible options for medical personnel to acquire and apply medical knowledge to improve the care and management of patients with HT. It also provides standardized learning methods and content for a larger cohort of medical students as it is accessible at all times [31, 32]. The study has some implications for future medical training in Uganda and other sub-Saharan countries, for considerations for blended learning with the requisite internet capabilities for medical schools. It also addresses gender-based research for examining learning behaviors to ensure female students benefit optimally.

We acknowledge several limitations. The study was exploratory in design to assess the effectiveness and acceptance of self-paced courses to enhance knowledge of medical students. A controlled design, with a difference in difference analysis would have provided stronger empirical evidence. Likewise, the effect size selected was 0.5 based on programmatic interest to improve knowledge competencies for health systems strengthening. Funding limitations and available pool of 3–5 year medical students, was a constraining factor to select a higher sample size and stronger effect size. The study was not implemented with program managers and facility supervisors who are the primary audience for the online course, as they provide oversight to the planning and operations of HT control programs, presenting another study limitation. Due to the prevailing COVID-19 pandemic-related constraints, including frequent university closures and lock downs, alternative measures had to be instituted for internet access, and frequent reminders were needed to encourage the completion of the post test. Lack of time to take the tests, length of test, and unreliable internet connectivity were frequently mentioned as reasons for dropping out of the study. However, the study highlights important findings to improve knowledge capacities of medical students who will eventually manage HT.

## Conclusion

Provision of high quality healthcare warrants integrated strategies to complement conventional training methods that are the norm [33]. Blended learning platforms integrating inexpensive and accessible self-paced online course technology innovations with the traditional lecture and clinical rotations have the potential to improve the knowledge skills of medical students in resource-constrained healthcare settings, where opportunities for training remain scarce or expensive.

## Supporting information

S1 Checklist Inclusivity in global research. (DOCX)

S1 Fig Distribution of length of time between pre and post test completion. (TIF)

S2 Fig Distribution of length of time between pre and post test completion–By gender. (TIF)

S3 Fig Distribution of length of time between pre and post test completion–By medical school year. (TIF)

S1 Table Pre-post hypertension knowledge scores by concept. (DOCX)

S1 Data (DTA)

## Abbreviations

BP: Blood Pressure
DBP: Diastolic Blood Pressure
HT: Hypertension
LMIC: Low and Middle Income Countries
NCD: Non Communicable Diseases
NGO: Non-Governmental Organization
SBP: Systolic Blood Pressure
