# Supplementary figures and images for: Self-paced online learning to improve knowledge competencies for hypertension among medical students in Uganda: A pre-post study
Source: PLOS Glob Public Health. 2023 Jul 17;3(7):e0001609. doi: 10.1371/journal.pgph.0001609 (PMC10351720; doi:10.1371/journal.pgph.0001609)

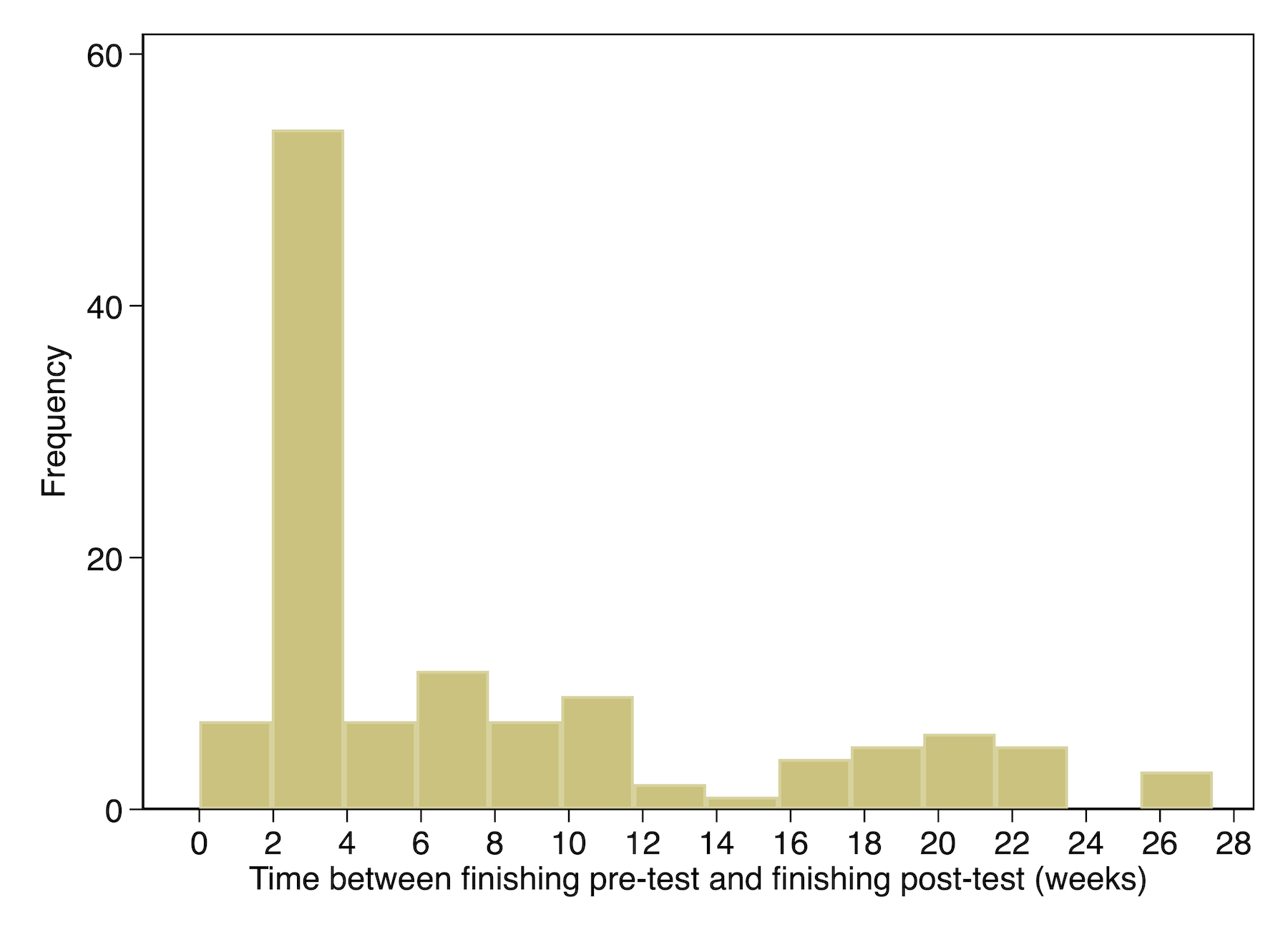

Supplement: S1 Fig — (TIF) [file pgph.0001609.s002.tif]

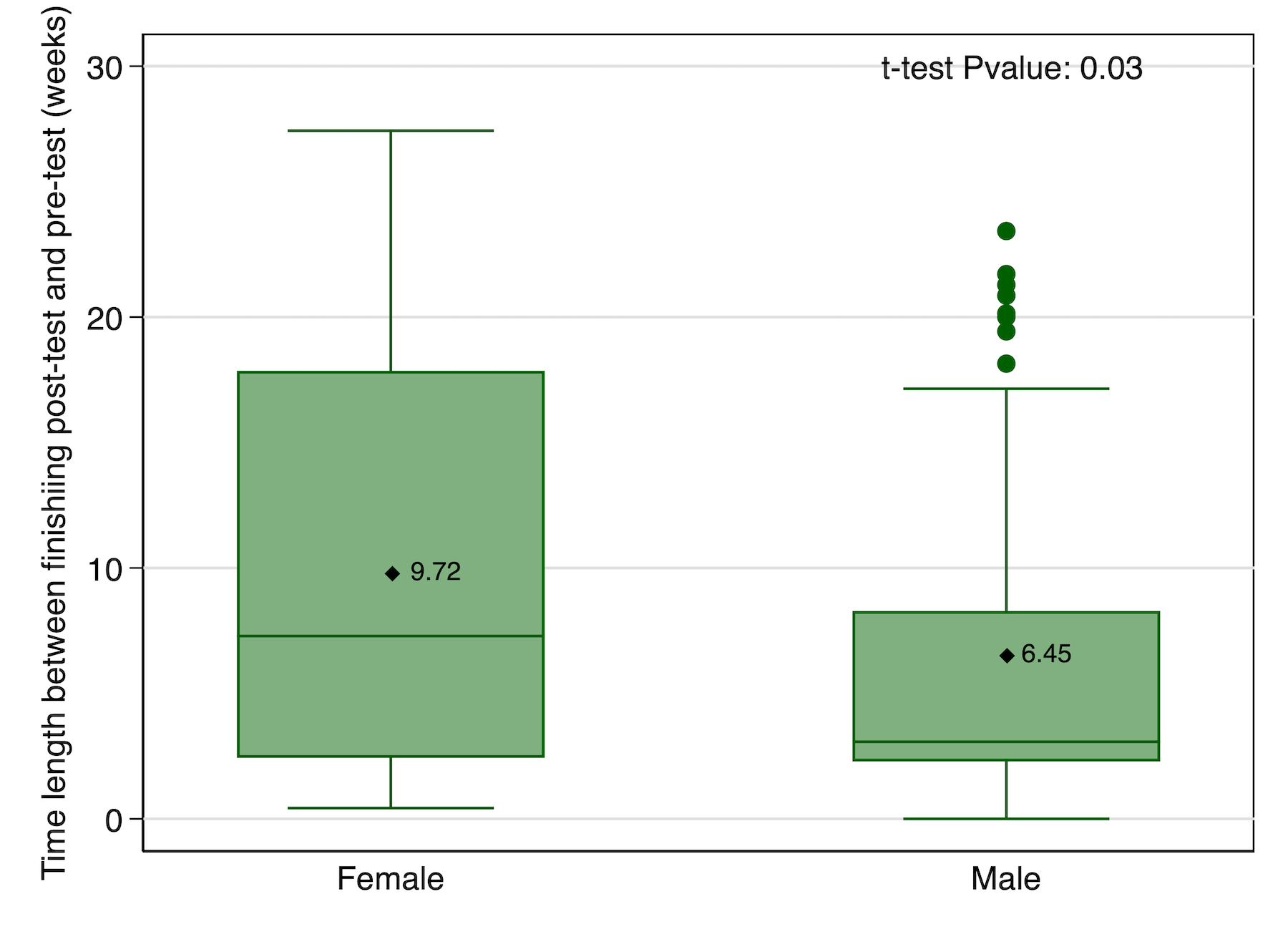

Supplement: S2 Fig — (TIF) [file pgph.0001609.s003.tif]

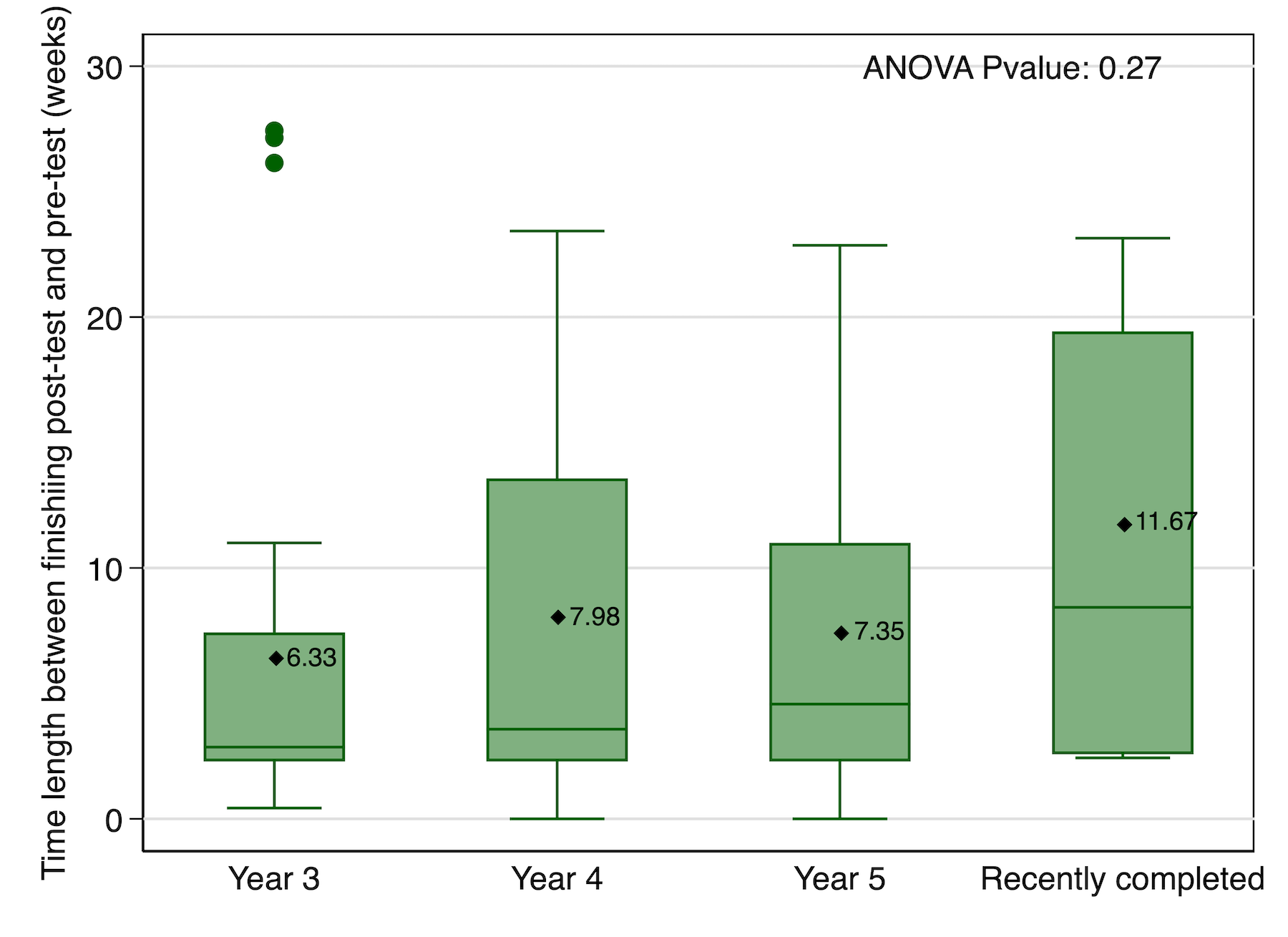

Supplement: S3 Fig — (TIF) [file pgph.0001609.s004.tif]
