# Supplementary material for: Self-paced online learning to improve knowledge competencies for hypertension among medical students in Uganda: A pre-post study
Source: PLOS Glob Public Health. 2023 Jul 17;3(7):e0001609. doi: 10.1371/journal.pgph.0001609 (PMC10351720; doi:10.1371/journal.pgph.0001609)
Supplement: S1 Table — (DOCX) [file pgph.0001609.s005.docx]

## Table S1: Pre-Post Hypertension Knowledge Scores by Concept

| Component | Pre-test Score  (Mean ± SD) | Post-test Score  (Mean ± SD) | Post-pre difference | p-value^a^ |
| --- | --- | --- | --- | --- |
| ***Module 1 Fundamentals of HT*** |  |  |  |  |
| **Concepts of HT** |  |  |  |  |
| Risk factors of HT | 0.94±0.23 | 0.98±0.16 | 0.03 | 0.158 |
| HT prevalence | 0.98±0.16 | 0.96±0.20 | -0.02 | 0.482 |
| Measuring and recording BP | 0.93±0.25 | 0.93±0.26 | -0.01 | 0.764 |
| Definition of HT | 0.92±0.28 | 0.98±0.13 | 0.07 | 0.020 |
| Common symptoms of HT | 0.19±0.39 | 0.30±0.46 | 0.11 | 0.019 |
| Global burden of HT | 0.41±0.49 | 0.69±0.47 | 0.27 | <0.001 |
| Session total | 4.37±0.80 | 4.83±0.89 | 0.45 | <0.001 |
| **Treatment of HT** |  |  |  |  |
| Treatment goal SBP/DBP 140/90mmHg | 0.67±0.47 | 0.72±0.45 | 0.05 | 0.368 |
| Lifestyle modification to reduce BP | 0.93±0.26 | 0.98±0.16 | 0.05 | 0.057 |
| Treatment with medication | 0.88±0.32 | 0.97±0.18 | 0.08 | 0.012 |
| 4-types of BP-lowering medication | 0.59±0.49 | 0.87±0.34 | 0.28 | <0.001 |
| Patient education on BP-lowering medicines | 0.84±0.37 | 0.96±0.20 | 0.12 | 0.001 |
| Session total | 3.91±1.02 | 4.49±0.63 | 0.58 | <0.001 |
| **HT-related complications** |  |  |  |  |
| Major complications of HT | 1.00±0.00 | 0.98±0.13 | -0.02 | 0.158 |
| Risks of uncontrolled HT | 0.29±0.46 | 0.66±0.48 | 0.37 | <0.001 |
| Family history of HT– risk of HT | 0.78±0.42 | 0.70±0.46 | -0.07 | 0.118 |
| Risks of high SBP/DBP | 0.71±0.46 | 0.82±0.39 | 0.11 | 0.027 |
| Session total | 2.78±0.86 | 3.17±0.87 | 0.39 | <0.001 |
| **Challenges of HT management** |  |  |  |  |
| Routine screening for HT | 0.90±0.30 | 0.94±0.23 | 0.04 | 0.198 |
| Challenges in diagnosis and management of HT | 0.94±0.23 | 0.98±0.13 | 0.04 | 0.058 |
| Factors that do not contribute to uncontrolled HT | 0.88±0.33 | 0.92±0.28 | 0.04 | 0.132 |
| Appropriate measurement of BP | 0.97±0.18 | 0.98±0.13 | 0.02 | 0.319 |
| Session total | 3.69±0.62 | 3.83±0.51 | 0.14 | 0.006 |
| **HT control programs** |  |  |  |  |
| Strategies for HT control programs | 0.98±0.13 | 0.98±0.16 | -0.01 | 0.566 |
| HT strategies to ensure medication adherence | 0.94±0.23 | 0.97±0.18 | 0.02 | 0.319 |
| Recommended strategies for HT control programs | 0.97±0.18 | 0.97±0.18 | 0.00 | 1.000 |
| Outcomes of effective HT control programs | 0.98±0.13 | 0.93±0.26 | -0.06 | 0.019 |
| CHW patient counseling to support medication adherence | 0.87±0.34 | 0.94±0.23 | 0.07 | 0.020 |
| Risk factors HT | 0.93±0.25 | 0.91±0.29 | -0.02 | 0.441 |
| Definition of HT | 0.59±0.49 | 0.74±0.44 | 0.15 | <0.001 |
| Lifestyle modification for limiting salt intake | 0.90±0.30 | 0.93±0.26 | 0.02 | 0.368 |
| Session total | 7.17±0.95 | 7.35±0.93 | 0.18 | 0.044 |
| Module 1 Mean Score | 21.91±2.52 | 23.65±2.51 | 1.74 | <0.001 |
|  |  |  |  |  |
| ***Module 2 Basics of HT Management*** |  |  |  |  |
| **Measuring blood pressure** |  |  |  |  |
| Confirming HT for patients in pain | 0.93±0.25 | 0.97±0.18 | 0.03 | 0.103 |
| Patient preparation prior to BP measurement | 0.81±0.39 | 0.93±0.26 | 0.12 | 0.006 |
| Sequence of steps for patient preparation prior to BP measurement | 0.40±0.49 | 0.63±0.49 | 0.22 | <0.001 |
| Patient texting can cause error in BP measurement | 0.44±0.50 | 0.59±0.49 | 0.15 | 0.009 |
| Standards for multiple BP measurements | 0.34±0.48 | 0.68±0.47 | 0.34 | <0.001 |
| Standards for BP measurement in emergency room | 0.74±0.44 | 0.75±0.43 | 0.01 | 0.870 |
| Regular monitoring of BP for normal patients | 0.24±0.43 | 0.28±0.45 | 0.04 | 0.386 |
| Precautions for patient prior to BP measurement | 0.86±0.35 | 0.93±0.25 | 0.07 | 0.028 |
| Standards for appropriate BP cuff placement | 0.45±0.50 | 0.45±0.50 | 0.01 | 0.867 |
| Session total | 5.21±1.35 | 6.21±1.50 | 0.99 | <0.001 |
| **Devices to measure blood pressure** |  |  |  |  |
| Advantages of automated BP devices | 0.64±0.48 | 0.74±0.44 | 0.11 | 0.047 |
| Terms used for automated and aneroid device | 0.19±0.39 | 0.55±0.50 | 0.36 | <0.001 |
| Session total | 0.83±0.57 | 1.29±0.72 | 0.46 | <0.001 |
| **HT diagnosis** |  |  |  | <0.001 |
| Protocol for initiating HT treatment | 0.69±0.47 | 0.83±0.38 | 0.14 | 0.004 |
| Protocol for return visit for patient with elevated BP | 0.68±0.47 | 0.56±0.50 | -0.12 | 0.023 |
| Session total | 1.36±0.66 | 1.39±0.64 | 0.02 | 0.711 |
| **Treatment options for HT** |  |  |  |  |
| BP medication that does not require lab monitoring | 0.36±0.48 | 0.67±0.47 | 0.31 | <0.001 |
| Protocol for determining type of medication | 0.34±0.48 | 0.48±0.50 | 0.14 | 0.013 |
| Factor for protocol selection | 0.18±0.39 | 0.18±0.39 | 0.00 | 1.000 |
| Protocol for all types of 1^st^ line BP medication | 0.57±0.50 | 0.89±0.31 | 0.32 | <0.001 |
| Recommended food for dietary modification | 0.78±0.42 | 0.81±0.39 | 0.03 | 0.482 |
| First line treatment option for HT | 0.19±0.39 | 0.45±0.50 | 0.26 | <0.001 |
| Advantage of single treatment protocol | 0.65±0.48 | 0.70±0.46 | 0.05 | 0.357 |
| Effect of quitting smoking for patients with HT | 0.47±0.50 | 0.55±0.50 | 0.07 | 0.171 |
| Advantages of fixed dose combination | 0.51±0.50 | 0.49±0.50 | -0.02 | 0.682 |
| HT treatment options | 0.60±0.49 | 0.60±0.49 | -0.01 | 0.880 |
| BP lowering medication options | 0.36±0.48 | 0.67±0.47 | 0.31 | <0.001 |
| Safety and risk of side effects of BP medications | 0.27±0.45 | 0.55±0.50 | 0.28 | <0.001 |
| Treatment options in contexts with no lab facilities | 0.74±0.44 | 0.83±0.37 | 0.10 | 0.051 |
| Reason for automated BP device in resource limited contexts | 0.69±0.47 | 0.83±0.38 | 0.14 | 0.005 |
| 1^st^ line of BP medicines | 0.79±0.41 | 0.93±0.26 | 0.13 | 0.001 |
| Session total | 7.14±2.07 | 8.96±2.28 | 1.82 | <0.001 |
| Module 2 score | 14.90±3.30 | 18.51±4.25 | 3.61 | <0.001 |
|  |  |  |  |  |
| ***Total score of modules 1 and 2*** | 36.81±4.75 | 42.17±6.11 | 5.36 | <0.001 |

^a^ *Paired t tests*
